# Factors influencing and long-term effects of manual myotomy phenomenon during physiotherapy for congenital muscular torticollis

**DOI:** 10.1186/s12891-022-05788-7

**Published:** 2022-10-01

**Authors:** Zhenhui Zhao, Hansheng Deng, Xin Qiu, Gen Tang, Huijia Zheng, Fang Yang, Futang Gao, Zhengyu Wu, Yuanheng Li, Shuaidan Zeng, Jiaxin Zhao, Yiyuan Sun, Ziheng Zhou, Yu Tang, Zhiwen Cui, Weiqing Li, Xiaodi Chen, Ting Cai, Xian Liu, Shicheng Li, Qisong Yang, Shengping Tang, Zhu Xiong

**Affiliations:** 1 Shenzhen Children’s Hospital of China Medical University, Shenzhen, People’s Republic of China; 2 Chengdu 363 Hospital of Southwest Medical University, Chengdu, Sichuan Province People’s Republic of China; 3 Maternity and Children Health Care Hospital of Luohu District, Shenzhen, Guangdong Province People’s Republic of China; 4 Shenzhen Baoan Women’s and Children’s Hospital, Shenzhen, Guangdong Province People’s Republic of China; 5 Hefei Cancer Hospital, Chinese Academy of Science, Hefei, People’s Republic of China; 6 Shenzhen Institutes of Advanced Technology, Chinese Academy of Sciences, Shenzhen, Guangdong Province People’s Republic of China; 7 Guangxi Medical University, Nanning, Guangxi Province People’s Republic of China; 8 Department of Pediatric Orthopedics, Shenzhen Children’s Hospital, Shenzhen, Guangdong China

**Keywords:** Manual myotomy, Congenital muscular torticollis, Physiotherapy, Infant, Sternocleidomastoid muscle

## Abstract

**Purpose:**

To investigate the factors influencing and long-term effects of manual myotomy (MM) occurring during physiotherapy for congenital muscular torticollis (CMT).

**Methods:**

We retrospectively collected the clinical data of children with CMT receiving physiotherapy between 2008 and 2018. The children were divided into manual myotomy (MM) and non-manual myotomy (NMM) groups according to whether MM occurred during treatment. We assessed physiotherapy outcomes in children with CMT using craniofacial asymmetry parameters and the Cheng–Tang rating score. By measuring the ear-eye distance, ear-nose distance, eye-mouth distance, ear-mouth distance, half-head circumference, and half-head top at two sides to evaluate craniofacial asymmetry. Based on the Cheng–Tang assessment criteria, we recorded the range of rotation, range of lateral flexion, the status of the contracted muscle, the hardness of the mass, the extent of head tilting during activities and sleeping, the status of daily activities, face size, type of head shape, cranial changes, and subjective head tilting to assess the effectiveness of treatment. Clinical data and outcome indicators (craniofacial asymmetry parameters and Cheng–Tang rating score) were compared.

**Results:**

The MM group had a significantly higher total Cheng–Tang rating score than the NMM group (*P* < 0.05). Age at initial physiotherapy session was the risk factor for MM during physiotherapy.

**Conclusion:**

Children with CMT developing MM during physiotherapy generally have a good outcome, although we do not recommend MM as a goal of treatment. Physiotherapists should understand this phenomenon, assess relevant factors to predict risk, and carefully observe treatment to prevent possible complications.

**Supplementary Information:**

The online version contains supplementary material available at 10.1186/s12891-022-05788-7.

## Introduction

### Background

Congenital muscular torticollis (CMT) is a common congenital musculoskeletal disease [1–4]. It can be accompanied by developmental dysplasia of the hip (DDH), with an incidence of 0–29%[5, 6]. The reported incidence of CMT varies from less than 1% to 3.92%, but can also be as high as 16% [1–4, 7–10]. CMT typically occurs in infants two weeks after birth and manifests as a mass in the sternocleidomastoid muscle (SCM) or head tilting and restricted neck rotation due to SCM contracture [1, 4, 17]. The pathogenic mechanism of CMT remains controversial, with theories including birth trauma, abnormal fetus position, infection [2, 4, 11–13], and SCM dysplasia [4, 14]. Usually, fibrotic SCM contracture causes the head to tilt to the affected side, the chin to turn towards the unaffected side [1, 4, 8, 15, 16], limited neck rotation to the affected side, and lateral flexion to the unaffected side [15]. Since the disease worsens without treatment, infants can develop facial asymmetry, and cranial and spinal deformity [16].

Treatment for CMT includes conservative and surgical treatment [11, 12, 17, 18]. Physiotherapy is an effective and stable conservative approach for infants under one year old [19], and achieves a good outcome in 60-90% of infants [18]. Surgery is considered when physiotherapy fails to resolve the symptoms after more than six months of continuous treatment [12, 17]. During physiotherapy, some infants may experience a partial or complete rupture of the SCM, with marked improvement in the range of rotation and lateral flexion in the neck, which is recognized as manual myotomy (MM) [18, 20, 21]. MM occasionally occurs in the process of physical therapy for CMT, with a reported incidence of 8%–9%, but its mechanism and effects on prognosis remain unclear [1, 18]. Complications may arise and may even raise concerns for therapists and guardians [29].

### Objectives

However, it is still unclear how MM affects the long-term prognosis of infants with CMT, and which factors are related. Moreover, some physical therapists lack the knowledge of how to predict, recognize and treat unexpected MM in physiotherapy. Consequently, they may not provide appropriate treatment. The study objectives are 1) to explore the occurrence of MM in children with CMT during physiotherapy, 2) to explore its impact on the clinical course and long-term prognosis of children, 3) to analyze the risk factors of MM, and 4) to better guide the physiotherapy of infants and young children with CMT.

## Methods

### Study design

**Fig. 1:**
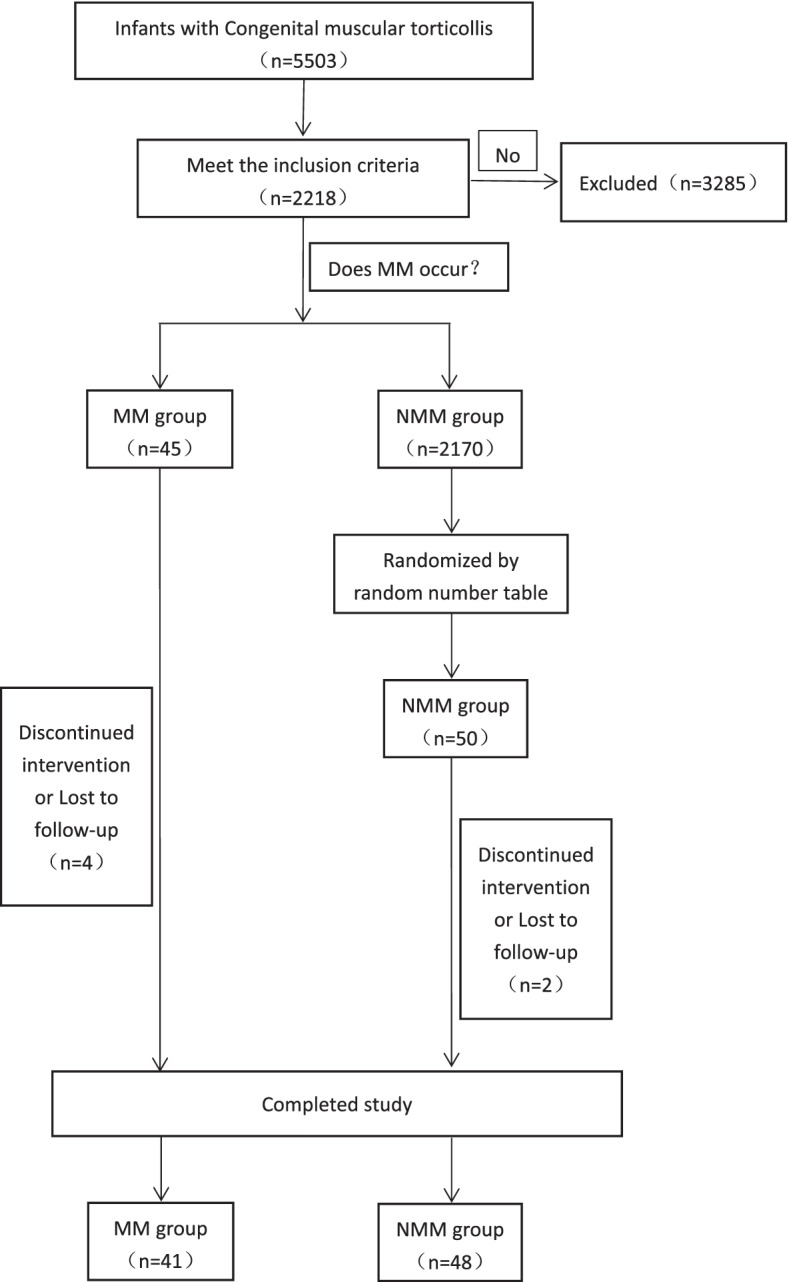
Overall flow chart

We retrospectively collected the clinical data of children with CMT receiving physiotherapy between 2008 and 2018. The children were divided into manual myotomy (MM) and non-manual myotomy (NMM) groups according to whether MM occurred during treatment. We assessed physiotherapy outcomes in children with CMT using craniofacial asymmetry parameters and the Cheng–Tang rating score. Through the inclusion and exclusion criteria, a total of 89 participants in the two groups were finally included. Their clinical data and efficacy evaluation indicators were obtained through long-term follow-up. Finally, we used appropriate statistical methods to compare the clinical data and efficacy evaluation indicators of the two groups and draw corresponding conclusions based on clinical practice. (Fig. 1).

### Participants

#### Inclusion and exclusion criteria.

The inclusion criteria were [1, 19]: (a) typical clinical manifestations: a history of head tilting and a neck mass; signs of head tilting to the affected side with the chin turning towards the unaffected side, limited neck rotation to the affected side, and restricted lateral flexion of the neck to the normal side; and palpable tightness or thickening of the SCM; (b) findings of SCM abnormalities on neck ultrasound; (c) completing the whole course of standardized treatment after initial diagnosis at our hospital.

The exclusion criteria were [1, 19]: (a) ocular torticollis, vestibular torticollis, osseous torticollis, or torticollis due to nervous system disorders, neck infection or inflammation, or other diseases; (b) failure to follow up.

### Study population

We enrolled 89 infants with CMT who received physiotherapy at the Department of Torticollis, Shenzhen Children’s Hospital from February 2008 to January 2018. During the study period, a total of 45 infants with CMT developed MM during physiotherapy. Of those, 41 had complete clinical data and were included in the MM group. During the same period, we enrolled 48 infants with CMT using the random number table method, who received physiotherapy but did not develop MM into the NMM group. Both groups of infants were followed up by telephone or at the clinic to obtain clinical data. All infants were given standard physical treatment. This study was approved by the ethics committee of the hospital. The guardians were informed of the objective and content of the study and provided voluntary informed consent(Fig. 1).

### Definition of MM

**Fig. 2:**
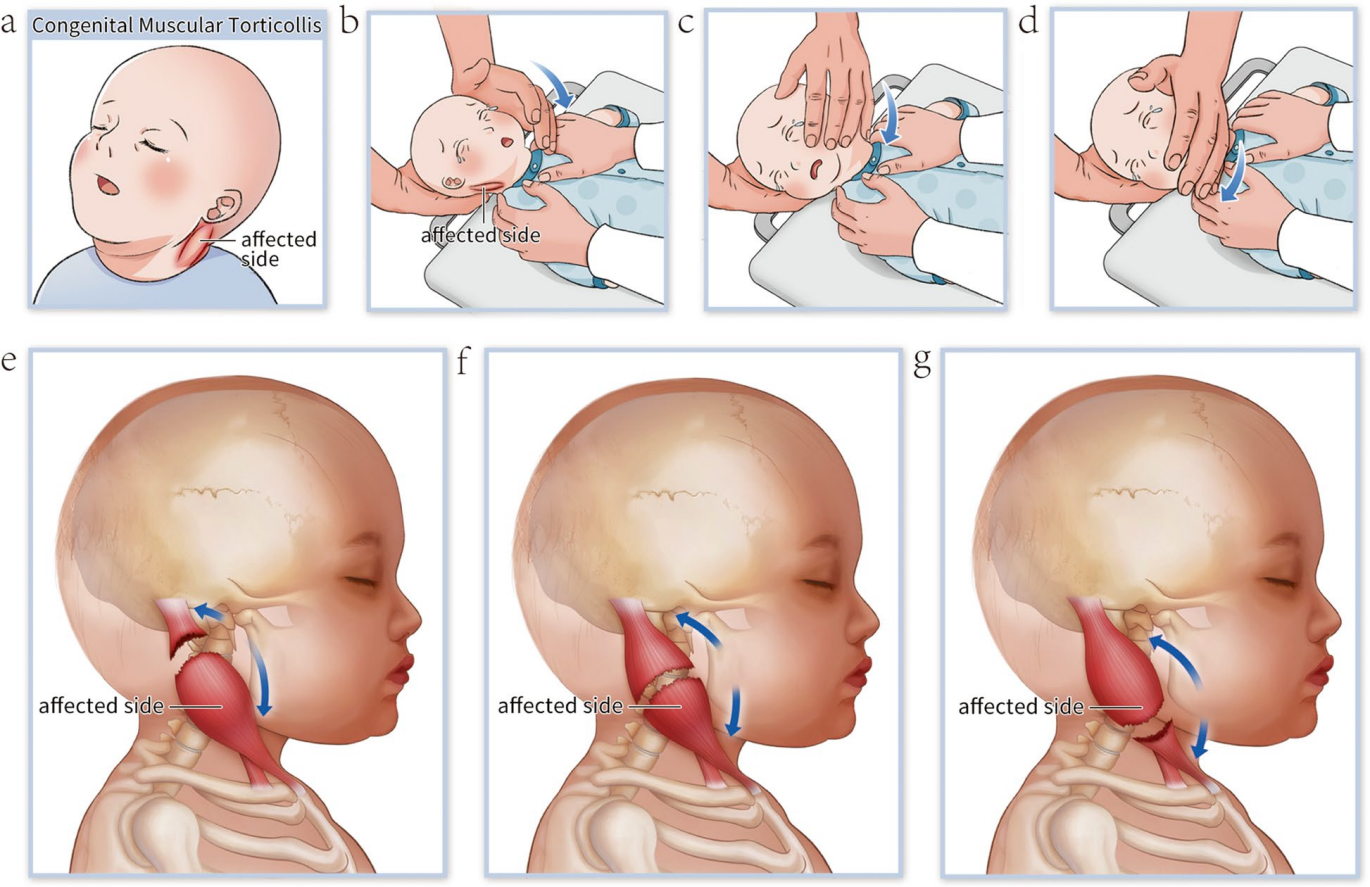
Schematic diagram of physiotherapy manipulation for infants with CMT, and SCM changes after MM (**a**) Clinical manifestations in infants with CMT (**b-d**) Physiotherapy manipulation (**e-g**) Three types of changes in the SCM after MM Note: There is no consensus on whether children with CMT have incomplete or complete rupture of the SCM after developing MM during physical therapy in children with CMT. In order to show more clearly and directly where the ruptured area of the SCM is in the SCM mass after MM, we marked the SCM as completely ruptured in the figure

According to relevant literature[4, 18, 20, 21], we defined MM as the occurrence of a partial or complete rupture of the SCM during the following physical therapies for CMT: manual stretching with intentionally accelerated speed, manual stretching with unintentionally accelerated speed, and conventional manual stretching with constant speed (Fig. 2).

### Treatment

Both groups were treated by trained physiotherapists. Before treatment, relevant clinical information was carefully obtained.

Physical therapy protocol was as follows [1, 2, 9, 17, 19]: the infant lay supine on a treatment table without a pillow. Standing on the cephalic side of the infant, the physiotherapist pinched the infant’s bilateral SCMs with both hands, and gently performed passive rotation and lateral flexion of the head and neck to assess neck mass, the range of motion of the neck, and the extent of limitation of motion on the affected side. Skin oil could be applied on the affected SCM to protect the skin. With the hands on the chin and the occipital area of the normal side, the physiotherapist stretched and rotated the chin to the shoulder of the affected side at an even pace with contralateral force of both hands. When reaching the maximum degree of rotation, the physiotherapist used the forearm to resist against the chin to fix it in position for 2–3 s(10–12 times per minute). For every 5–10 stretches, the physiotherapist pinched the site of the local contracture of the SCM to detect any changes. The frequency of the physical therapy depended on the recovery of the infant, and the intensity of manipulation could be adjusted according to the severity of the disease. If the infant coughed or cried during treatment, the physiotherapist suspended the treatment and comforted the infant (Fig. 2). The whole process lasted 5–10 min.

### Efficacy assessment

Definition of clinical cure: (a) head tilt and contracture mass in the affected SCM disappeared, and the muscle became soft; (b) there was no substantial limitation in passive motion of the neck, and the difference in the degree of rotation of both sides was within 5°, without abnormalities after a follow-up of at least six months.

All participants were followed up regularly at the Torticollis Clinic, and were examined for craniofacial parameters. Based on the Cheng–Tang assessment criteria [17, 22, 30], we recorded range of rotation, range of lateral flexion, the status of the contracted muscle, the hardness of the mass, the extent of head tilting during activities and sleeping, the status of daily activities, face size, type of head shape, cranial changes, and subjective head tilting to assess the effectiveness of treatment. Each item above was rated as: excellent (three points), good (two points), fair (one point), or poor (zero), and the total Cheng–Tang rating score was calculated [17, 22, 30]. We also measured the ear-eye distance, ear-nose distance, eye-mouth distance, ear-mouth distance, half-head circumference, and half-head top at two sides to evaluate craniofacial asymmetry [22].

### Statistical methods

SPSS Version 19.0 was used for data analysis. Quantitative data were described as mean ± standard deviation and analyzed using the *t* test. Qualitative data were described as percentages and were analyzed using the chi-squared test or the Fisher’s exact test. A univariate logistic regression analysis was used to screen for possible risk factors for MM. With the significant factors from the univariate analysis as the independent variables (*P* value for inclusion was set at 0.05 and the value for removal at 0.10), a non-conditional logistic regression analysis was performed to further identify risk factors for MM. *P* < 0.05 was considered statistically significant.

## Results

### Comparison of general clinical data between the MM and NMM groups

A total of 89 infants with CMT who received physiotherapy from February 2008 to January 2018 were included, with 41 cases in the MM group and 48 in the NMM group. The mean length of follow-up was 67.08 ± 34.65 months.

In the MM group, the infants were aged 31 ± 22.35 days (range: 3–115 days) at onset of MM, and 90.24% of the cases developed MM during the first physical therapy session. There were 26 boys (63.41%) and 15 girls (36.59%). The torticollis was on the right side in 23 cases (56.10%). There were 38 infants (92.68%) with normal birth weight, 1 (2.44%) with low birth weight, and 2 cases (4.88%) of fetal macrosomia. The percentage of first delivery was 75.61%. Most cases had normal amniotic fluid status, antepartum fetal movement, and fetal position; in one case (2.44%) the mother had low amniotic fluid, and one case (2.44%) had abnormal antepartum fetal movement. A total of 13 infants (31.71%) were born with breech presentation, and one (2.44%) with foot presentation. Twenty-two infants (53.66%) were born through vaginal delivery, and 19 (46.34%) through caesarean section. Seven infants (17.07%) had a nuchal cord at birth. There was one case (2.44%) each of vacuum-assisted delivery and oxytocin-assisted delivery. Eight cases (19.51%) were born with DDH. Four infants (9.76%) had a family history of CMT.

In the NMM group, there were 24 boys (50%) and 24 girls (50%). The torticollis was on the right side in 27 cases (56.25%) and on the left side in 21 cases (43.75%). There were 44 infants (91.67%) with normal birth weight, 2 (4.17%) with low birth weight, and 2 cases (4.17%) of fetal macrosomia. The percentage of first delivery was 66.67%. The majority of cases had normal amniotic fluid status, antepartum fetal movement, and fetal position; in four cases (8.33%) the mothers had low amniotic fluid, and in three cases (6.25%) there was abnormal antepartum fetal movement. Eleven infants (22.92%) were born with breech presentation, and two (4.17%) with foot presentation. Twenty-nine infants (60.42%) were born through vaginal delivery, and 19 (39.58%) through caesarean section. Five infants (10.42%) had a nuchal cord at birth. Two infants (4.17%) were delivered with forceps assistance, and one (2.08%) with vacuum assistance. Three cases (6.25%) were born with DDH. Six infants (12.50%) had a family history of CMT.

At baseline, no significant differences (*P* > 0.05)were found in sex, side of involvement, perinatal data, and family history between the MM and NMM groups (Table 1).

**Table 1:** Comparison of general clinical data between MM group and NMM group

Variables	MM group number (%)	NMM group number (%)	*P*
Sex			0.204
Male	26 (63.41%)	24 (50%)	
Female	15 (36.59%)	24 (50%)	
Side of involvement			0.988
Left	18 (43.90%)	21 (43.75%)	
Right	23 (56.10%)	27 (56.25%)	
Birth weight			0.488
Low weight	1 (2.44%)	2 (4.17%)	
Normal weight	38 (92.68%)	44 (91.67%)	
Fetal macrosomia	2 (4.88%)	2 (4.17%)	
Gravidity			0.464
1	26 (63.41%)	30 (62.50%)	
2	10 (24.39%)	11 (22.92%)	
3	3 (7.32%)	5 (10.42%)	
4	1 (2.44%)	1 (2.08%)	
5	1 (2.44%)	0 (0)	
6	0 (0)	1 (2.08%)	
Parity			0.396
1	31 (75.61%)	32 (66.67%)	
2	8 (19.51%)	14 (29.17%)	
3	1 (2.44%)	2 (4.17%)	
4	1 (2.44%)	0 (0)	
Status of amniotic fluid			0.369
Normal	40 (97.56%)	44 (91.67%)	
Low	1 (2.44%)	4 (8.33%)	
Antepartum fetal movement			0.621
Normal	40 (97.56%)	45 (93.75%)	
Abnormal	1 (2.44%)	3 (6.25%)	
Type of delivery			0.521
Vaginal delivery	22 (53.66%)	29 (60.42%)	
Cesarean section	19 (46.34%)	19 (39.58%)	
Delivery assistance methods			0.302
Forceps assistance	0 (0)	2 (4.17%)	
Vacuum assistance	1 (2.44%)	1 (2.08%)	
Oxytocin assistance	1 (2.44%)	0 (0)	
None	39 (95.12%)	45 (93.75%)	
Fetal position			0.394
Normal presentation	27 (65.85%)	35 (72.92%)	
Breech presentation	13 (31.71%)	11 (22.92%)	
Foot presentation	1 (2.44%)	2 (4.17%)	
Variables	MM group number (%)	NMM group number (%)	*P*
Condition of birth			0.359
Nuchal cord	7 (17.07%)	5 (10.42%)	
Normal	34 (82.93%)	43 (89.58%)	
Presence of DDH			0.058
Yes	8 (19.51%)	3 (6.25%)	
No	33 (80.49%)	45 (93.75%)	
Family history			0.683
Yes	4 (9.76%)	6 (12.50%)	
No	37 (90.24%)	42 (87.50%)	
Outcome			0.497
Referral to surgery	0 (0)	2 (4.17%)	
Cure	41 (100%)	46 (95.83%)	

### Comparison of treatment between MM and NMM groups

In the MM group, the infants were diagnosed with CMT at a mean age of 26.9 ± 17.34 days (range: 3–83 days), and received the first physiotherapy session at a mean age of 29.63 ± 21.85 days (range: 3–115 days). The total duration of physical treatment was 343.93 ± 309.31 days (range: 1–1404 days). The number of physiotherapy clinic visits was 20.85 ± 19.87 (range: 1–96). The time to SCM mass disappearance was 51.2 ± 96.14 days (range: 4–599 days). The total number of physiotherapy sessions was 41.95 ± 21.97 (range: 4–97). Thirty-two infants had pre-treatment ultrasound examination. The maximum thickness of the SCM mass was 34.88 ± 7.04 mm (range: 21–50 mm), and the volume of the SCM mass was 10,426.19 ± 4540.48 mm^3^ (range: 3000–18,750 mm^3^).

**Fig. 3:**
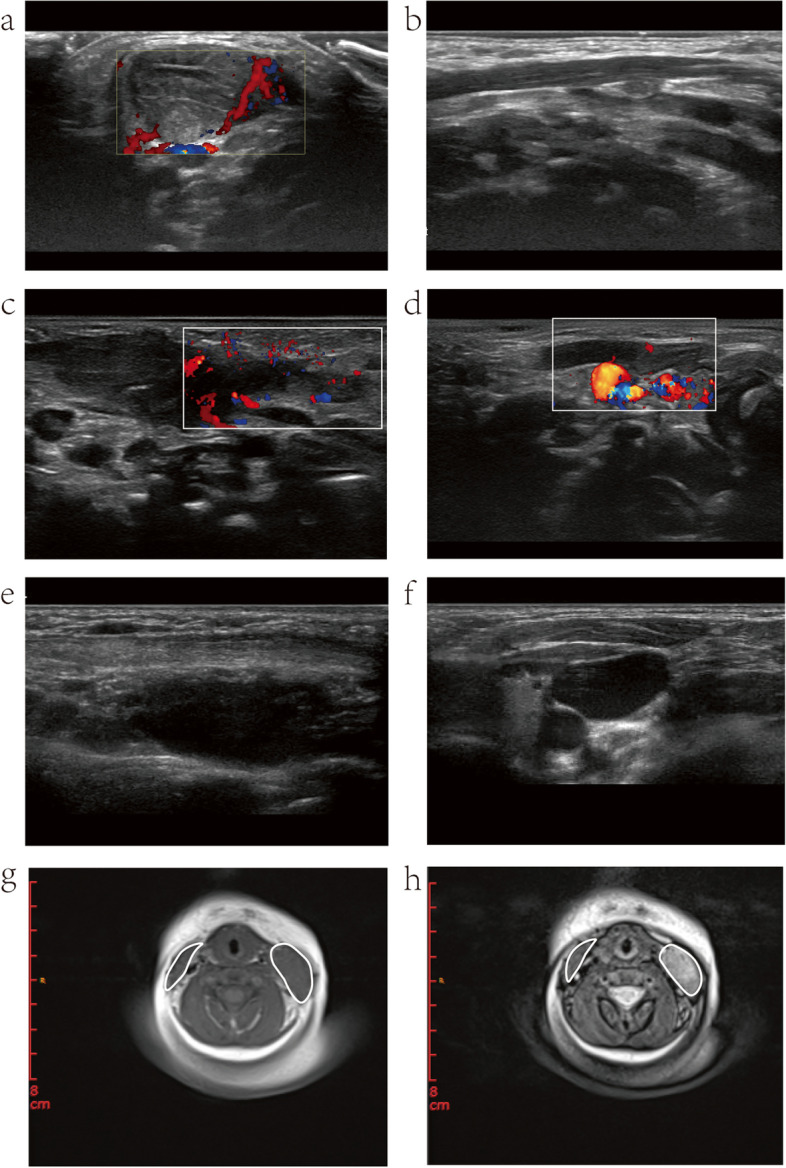
Ultrasound and MRI images of the infant in Case one at initial visit, immediately after MM, and at final follow-up (**a**) and (**b**) Ultrasound images of the involved and unaffected sides at initial visit (**c**) and (**d**) Instant ultrasound images of the involved and normal sides after MM (**e**) and (**f**) Ultrasound images of the involved and normal sides at final follow-up (**g**) Instant MRI-T1_tse images after MM (**h**) Instant MRI-T2_tse images after MM

In the NMM group, the infants were diagnosed with CMT at a mean age of 41.90 ± 21.02 days (range: 7–102 days), and received the first physiotherapy session at a mean age of 54.69 ± 55.65 days (range: 11–353 days). The total duration of physical treatment was 377.23 ± 297.85 days (range: 11–1152 days). The number of physiotherapy clinic visits was 27.5 ± 29.00 (range: 10–99). The time to SCM mass disappearance was 355.27 ± 195.16 days (range: 55–719 days). The total number of physiotherapy sessions was 54.46 ± 32.88 (range: 5–137). Thirty-one infants had pre-treatment ultrasound examination. Maximum thickness of the SCM mass was 31.13 ± 5.52 mm (range: 17–40 mm), and volume of the SCM mass was 9510.55 ± 5427.27 mm^3^ (range: 1785–29,400 mm^3^). (Fig. 3; Supplementary Fig. 1.

The independent samples *t*-tests for the above data showed that the MM group was significantly younger at diagnosis (mean age: 26.9 days *vs* 41.9 days, *P* < 0.05) and at first physiotherapy session (mean age: 29.63 days *vs* 54.69 days, *P* < 0.05) compared with the NMM group. The total number of physiotherapy sessions was significantly smaller in the MM group (mean total number: 41.95 *vs* 54.46, *P* < 0.05). The MM group had a significantly greater maximum thickness of the involved SCM (mean maximum thickness: 31.13 mm *vs* 34.88 mm, *P* < 0.05), a significantly greater thickness difference of the involved and normal SCMs (mean thickness difference: 25.15 mm *vs* 30.35 mm, *P* < 0.05), a significantly greater thickness ratio of the involved to normal SCM (mean thickness ratio: 7.65 *vs* 9.18, *P* < 0.05), and a significantly shorter time to SCM mass disappearance (mean time to mass disappearance: 355.27 days *vs* 51.2 days, *P* < 0.05). No significant differences were observed in the other factors (Table 2).

**Table 2:** Comparison of treatment between MM group and NMM group

Variables	MM group	NMM group	P
Age at diagnosis(day)	26.9 ± 17.34	41.90 ± 21.02	< 0.05
Age at initial physiotherapy session(day)	29.63 ± 21.85	54.69 ± 55.65	< 0.05
Time to SCM mass disappearance(day)	51.2 ± 96.14	355.27 ± 195.16	< 0.05
Total days of physiotherapy(day)	343.93 ± 309.31	377.23 ± 297.85	> 0.05
Number of physiotherapy clinic visits(times)	20.85 ± 19.87	27.5 ± 29.00	> 0.05
Total number of physiotherapy sessions(times)	41.95 ± 21.97	54.46 ± 32.88	< 0.05
Total physiotherapy frequency(times)	0.26 ± 0.31	0.22 ± 0.13	> 0.05
Maximum thickness of the involved SCM (mm)	34.88 ± 7.04	31.13 ± 5.52	< 0.05
Thickness difference of the involved and normal SCMs (mm)	30.35 ± 7.03	25.15 ± 9.07	< 0.05
Thickness ratio of the involved to normal SCM	9.18 ± 2.34	7.65 ± 2.69	< 0.05
SCM mass volume (mm^3^)	10,426.19 ± 4540.48	9510.55 ± 5427.27	> 0.05

No infants underwent surgery in the MM group, while two cases (4.2%) in the NMM group were finally referred to surgery.

### Comparison of outcome between the MM and NMM groups

Data on craniofacial asymmetry were available in 18 cases in the MM group and 15 cases in the NMM group. The two groups showed no significant differences in the six parameters of craniofacial asymmetry (ear-eye distance, ear-nose distance, eye-mouth distance, ear-mouth distance, half-head circumference, and half-head top; *P* > 0.05).

**Fig. 4:**
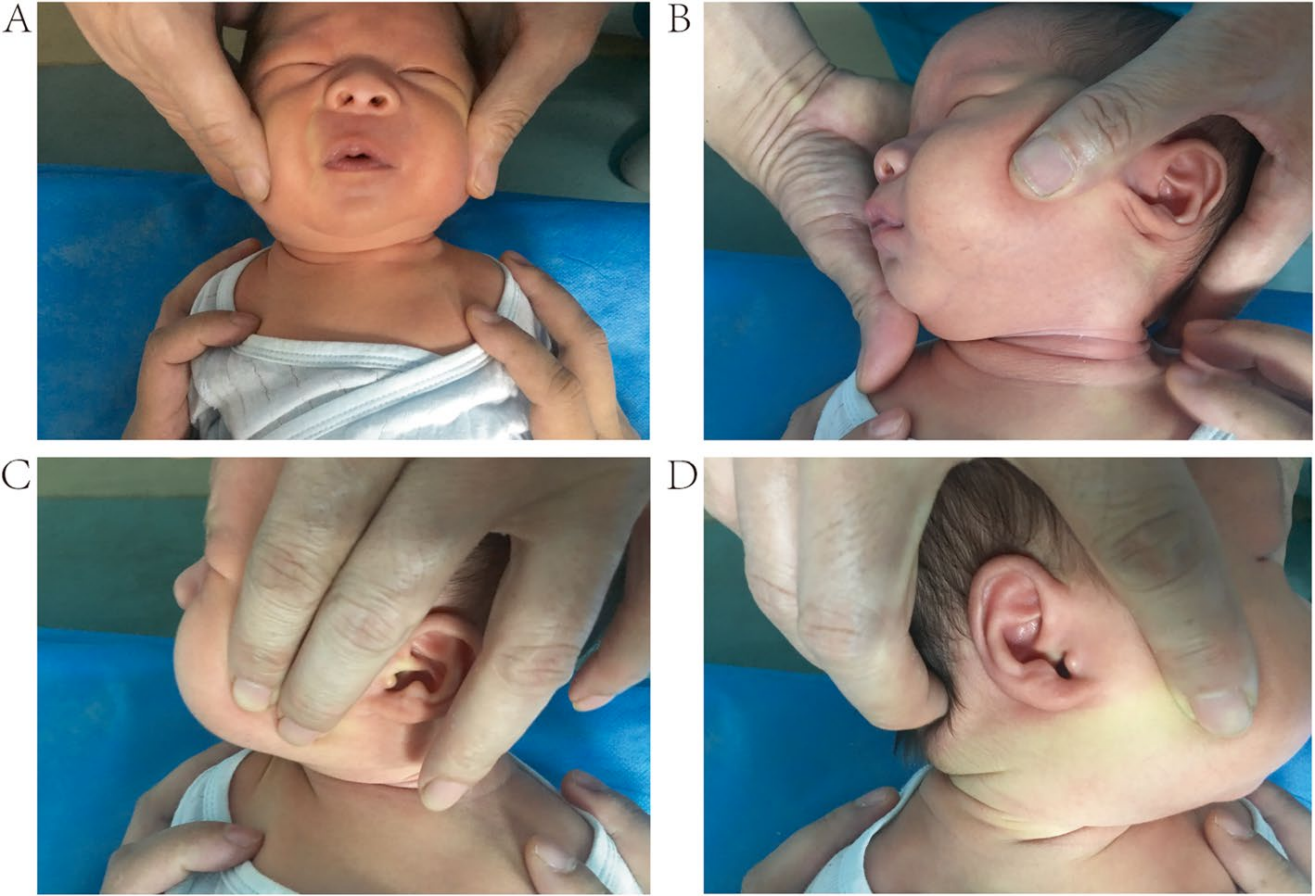
Physical examination of the infant in Case one after MM (**a**) Front view of the infant (**b**) View of the mass (**c**) View of neck rotation to the normal side (**d**) View of neck rotation to the involved side **Note:** the red triangle represents the location of the SCM mass

The total Cheng–Tang rating score and subjective head tilt were significantly different between the two groups (*P* < 0.05; Table 3; Fig.4; Supplementary Table1).

**Table 3:** Comparison of craniofacial asymmetry score and total Cheng-Tang rating score between MM group and NMM group

Item	Group	Number	Mean rank	Rank sum	*Z*	*P*	*M(P25-P75)*
Ear-eye distance difference	MM group	18	16.56	298.000	− 0.330	0.74	0 (0–0.50)
	NMM group	15	17.53	263.000			0.50 (0–0.50)
Ear-nose distance difference	MM group	18	16.25	292.500	− 0.548	0.584	0.25 (0–0.50)
	NMM group	15	17.9	268.500			0.50 (0–0.50)
Eye-mouth distance difference	MM group	18	16.5	297.000	− 0.385	0.7	0 (0–0.50)
	NMM group	15	17.6	264.000			0 (0–0.50)
Ear-mouth distance difference	MM group	18	17.83	321.000	− 0.656	0.512	0 (0–0.50)
	NMM group	15	16	240.000			0 (0–0.50)
Half-head circumstance difference	MM group	18	14.31	257.500	− 1.776	0.076	1.00 (0–1.50)
	NMM group	15	20.23	303.500			2.00 (0.500–3.00)
Half-head top difference	MM group	18	14.89	268.000	− 1.396	0.163	1.00 (0.375–2.50)
	NMM group	15	19.53	293.000			2.00 (1.00–3.0)
Total Cheng-Tang rating score	MM group	41	56.41	2313.000	− 4.318	< 0.05	32 (31–33)
	NMM group	48	35.25	1692.000			33 (32–35)

### Logistic regression analysis for risk factors

According to the univariate logistic regression analysis, the maximum thickness of the involved SCM, the thickness difference between the involved and normal SCMs, the thickness ratio of the involved to normal SCM, the presence of DDH, and age at initial physiotherapy were possible risk factors for the occurrence of MM (all *P* < 0.05). No significance was detected in SCM mass volume, fetal position, the presence of a nuchal cord or family history (all *P* > 0.05; Table 4). The non-conditional logistic regression analysis showed that age at initial physiotherapy session was the only risk factor for the occurrence of MM during physiotherapy in infants with CMT (*P* < 0.05; Table 5). The results showed that the risk of developing MM decreased by 0.937 for each additional day of age at initial physiotherapy session.

**Table 4:** Univariate analysis for possible risk factors

						95% confidence interval for Exp (B)	
Factor	β	SE	Wald	P	Exp (B)	Lower	Upper
Age at initial physiotherapy session(day)	-0.036	0.012	8.309	< 0.05	0.965	0.942	0.989
Maximum thickness of the involved SCM (mm)	0.097	0.044	4.772	< 0.05	1.102	1.010	1.202
SCM mass volume (mm^3^)	0.000	0.000	0.534	> 0.05	1.000	1.000	1.000
Thickness difference of the involved and normal SCMs (mm)	0.090	0.041	4.748	< 0.05	1.094	1.009	1.186
Thickness ratio of the involved to normal SCM	0.262	0.123	4.519	< 0.05	1.299	1.021	1.654
Breech delivery	-21.116	9220.898	0.000	> 0.05	0.000	0.000	-
Presence of DDH	-1.609	0.723	4.960	< 0.05	0.200	0.049	0.824
Nuchal cord	-0.638	0.655	0.947	> 0.05	0.528	0.146	1.909
Family history	0.323	0.747	0.187	> 0.05	1.381	0.319	5.973

**Table 5:** Multivariate analysis for possible risk factors

						95% confidence interval for Exp (B)	
Factor	β	SE	Wald	P	Exp (B)	Lower	Upper
Age at initial physiotherapy session(day)	-0.065	0.021	10.062	< 0.05	0.937	0.900	0.975
Presence of DDH	2.028	1.104	3.374	> 0.05	7.602	0.873	66.212
Maximum thickness of the involved SCM (mm)	-1.156	3.078	0.141	> 0.05	0.315	0.001	131.150
Thickness difference of the involved and normal SCMs (mm)	1.457	3.462	0.177	> 0.05	4.293	0.005	3795.911
Thickness ratio of the involved to normal SCM	-0.622	1.420	0.192	> 0.05	0.537	0.033	8.676

## Discussion

### The occurrence of MM.

Treatment approaches for CMT include observation only, physical therapy, neural and visceral manipulation, medicine injection, and surgery [17, 23, 24]. Early intervention for infants with CMT aims to prevent craniofacial deformity, limitations in neck motion, imbalance in muscle strength, and spinal deformity [1, 2]. For most infants with CMT, earlier professional and standardized treatment improves the outcome. Otherwise, SCM could develop into progressive fibrosis and contracture [4]. As the most commonly used treatment, physiotherapy may work by effectively stimulating myoblasts to produce normal myofibrils and thereby promote the regeneration and repair of the SCM [17, 25]. In addition, effective physical therapy allows the muscles of the neck to alternate between tension and relaxation, increasing the blood supply.

**Fig. 5:**
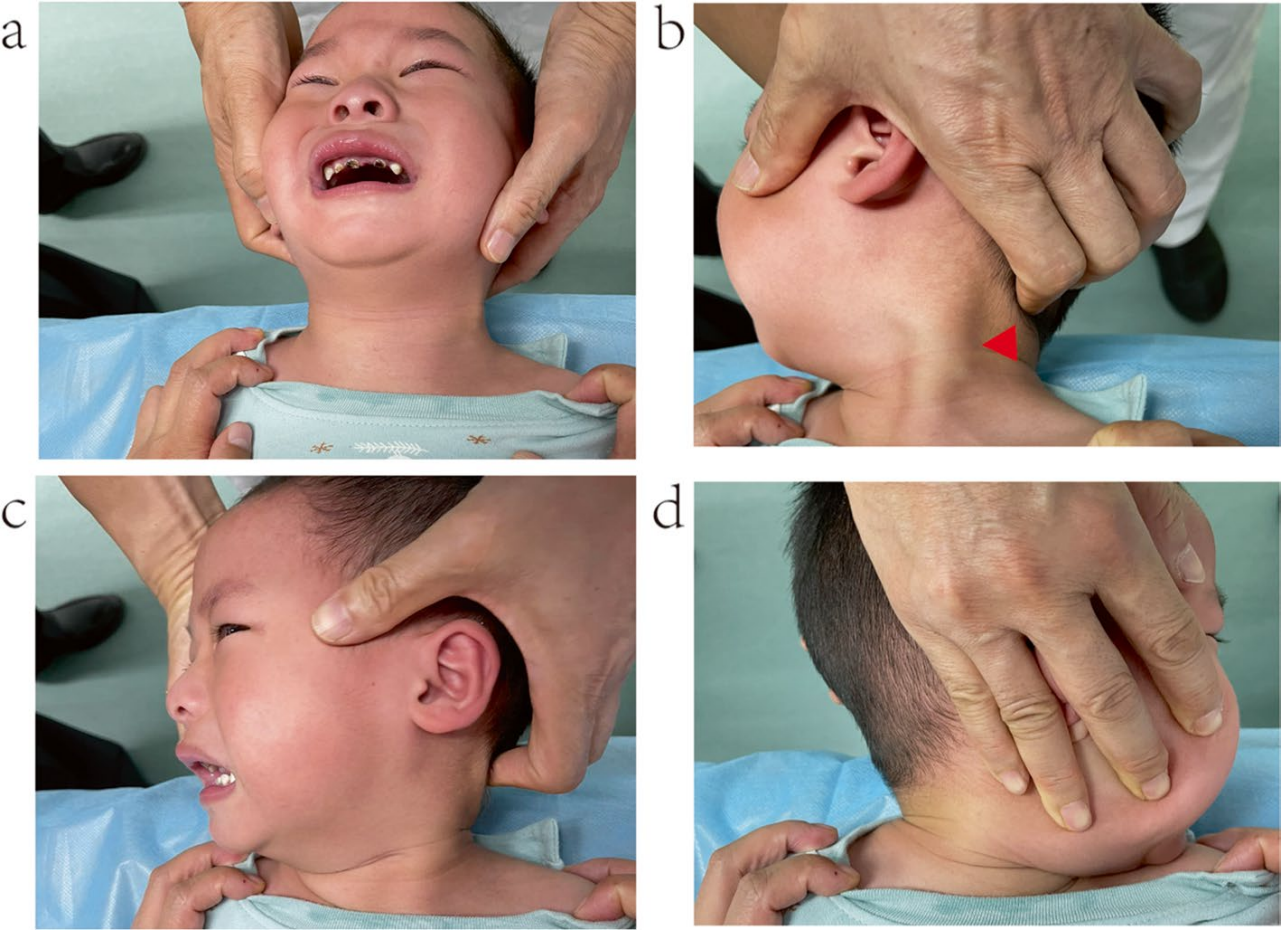
Physical examination of the infant in Case one at final follow-up (**a**) Front view of the infant (**b**) View of the mass (**c**) View of neck rotation to the normal side (**d**) View of neck rotation to the involved sideNote: the red triangle represents the location of the SCM mass

During physical treatment, the occurrence of MM in the SCM is not rare, with a reported incidence of 8%–9% [1, 18]. However, its definition and clinical manifestations are not clear. We defined it as a partial or complete rupture of the involved SCM during manual stretching of the head and neck with intentionally accelerated speed, manual stretching with unintentionally accelerated speed, and conventional manual stretching with constant speed. We described MM as a tetrad of signs: a snapping sound, an instant increase in the range of neck rotation, a shift of the SCM mass from above to below or from below to above, and enlargement of the SCM mass with a loss of SCM continuity on palpation (Fig. 4; Supplementary Fig.2). In our opinion, MM is a special clinical phenomenon in physical treatment, and not a deleterious event that may lead to poor outcome (Fig. 5).

#### Reasons for MM

The typical manifestation of CMT is the presence of a palpable mass in the substance of the contracted SCM [17]. According to previous literature[4, 18]and our clinical experience(Supplementary Material 1, case one and Supplementary Material 2, case two), infants with CMT usually present with a mass 1–3 cm in diameter around 15 days after birth, with a varying degree of hardness depending on the extent of SCM fibrosis [1, 4, 17]. Professional physiotherapy can reduce the size of the mass within one year, as well as reduce the tightness of the involved SCM. [1, 17, 25–27].

In early CMT, the muscle fibers of the SCM are disordered and include multiple cell types such as fibroblasts, myoblasts, adipocytes, and mesenchymal-like cells. The basic pathological changes of CMT are fibrosis, fat infiltration of the extracellular matrix (ECM), and decreased muscle fibers [14]. Transmission electron microscopy showed that the younger the child with CMT, the greater the extent of muscle fiber disarray and cell proliferation in the ECM [28]. Therefore, we speculate that, in early CMT, disorganized muscle fibers and multiple components in the ECM may lead to a disordered structure and decreased stability of the SCM, with a high risk of a rupture. In our study, the children developed MM at around 31 days, and approximately 90% of the events occurred in the first physiotherapy session. Age at initial physiotherapy session was the risk factor for the occurrence of MM during treatment. Our results support the above speculation in the context of pathological findings and transmission electron microscopy research.

In one case of CMT (Supplementary Material 1, case one), ultrasound and MRI images revealed a swollen and torn SCM with ring edema immediately after MM; and swelling in the mastoid portion of the SCM subsided on physical examination three days later, indicating an incomplete rupture of the SCM (Fig.3). However, due to a lack of dynamic MRI comparison before and after, it was unclear whether MM occurred in the proximal portion, the distal portion, or the mass itself. It was also unknown what microstructural changes happened in the SCM mass and the connection with the proximal and distal muscle tissues before and after MM.

#### Risk factors for MM

Cheng et al*.*[10] found a higher incidence of MM during physical treatment in children with CMT who presented earlier and with a more severe case, and where DDH was present. Kasai et al*. *[20]intentionally induced MM in children with CMT by increasing the stretching force, and observed that children with a larger SCM mass and more severe limitation of neck motion had a higher risk of MM during physiotherapy. They also found a higher frequency in infants around three weeks of age and a lower frequency among those over one month. In a study of 452 cases of SCM masses [17], approximately 8% of children developed MM during physiotherapy, and that it was more likely in children with DDH, left-side involvement, a rotation deficit of > 15°, and under one month old at presentation. In our study, a younger age at initial physiotherapy, a greater maximum thickness of the involved SCM, a greater thickness difference of the involved and normal SCMs, a higher thickness ratio of the involved to normal SCM, and the presence of DDH were associated with a higher risk of MM during treatment. The non-conditional logistic regression analysis demonstrated age at initial physiotherapy as the risk factor for MM. Our results were consistent with previous studies [10, 17, 20].

Therefore, this study indicated that physical therapists should carefully investigate relevant risk factors, inform the patient’s guardians of potential risk, and take preventive measures before manipulation.

#### Complications of MM and treatment

MM occasionally occurs in the process of physical therapy for CMT, but its mechanism and effects on prognosis remain unclear. Although patients will experience relaxation of the SCM and improvement of the range of neck rotation and side flexion after the event, they may develop complications such as hemorrhagic spots on the head and face, nausea and vomiting, local skin bruise or rupture, SCM swelling, skull base fracture, clavicle fracture, and cerebrospinal fluid otorrhea [29]. These may raise concerns for therapists and guardians. Inexperienced physiotherapists without standardized training often have no idea how to explain MM to guardians and what to do next. The preliminary conclusion of our research is that children with MM generally had a good outcome. Physiotherapists and guardians should not be overly concerned about the occurrence of MM during physical treatment. However, if MM does occur, physiotherapists should immediately stop the treatment, closely monitor the child’s vital signs, and examine the SCM mass for size, position, fluctuation, surrounding swelling, and ecchymosis.

#### Effects of MM on outcome

Cheng et al*. *[18]reported comparable response rates to physiotherapy for CMT in children with MM (95% of 41 cases) and without MM (90.7% of 404 cases), indicating similar therapeutic effects between children with and without MM. Furthermore, they observed that MM had no long-term adverse effects on children in a follow-up of 3.5 years. The authors explained that their research could not determine whether it was necessary to intentionally induce MM during physiotherapy. However, Shinoda et al. [21]thought that intentional induction of MM in physiotherapy was an effective treatment approach for CMT.

We found that the MM group was younger at diagnosis and initial physiotherapy than the NMM group, which suggested that children who were diagnosed and treated at an earlier time would be more likely to develop MM during physical treatment. Compared with the NMM group, the MM group had fewer physiotherapy sessions from the start to clinical cure, a shorter time to disappearance of the SCM mass, and a higher total Cheng–Tang rating score. No significant differences were observed in craniofacial asymmetry parameters. These results indicated that early recognition and treatment of CMT could help to improve therapeutic effects and shorten the course of treatment.

Nevertheless, based on our research and clinical experience, we do not think it necessary to induce MM with intentionally accelerated manipulation, as previous research suggested [20, 21]. We recommend that physiotherapists do not consider intentional induction of MM as a goal of treatment. They should understand and pay attention to this phenomenon to prevent or treat relevant complications. Studies to compare the efficacy of intentional manipulation with acceleration and conventional manipulation with constant speed are needed.

#### SCM changes after MM

**Fig. 6:**
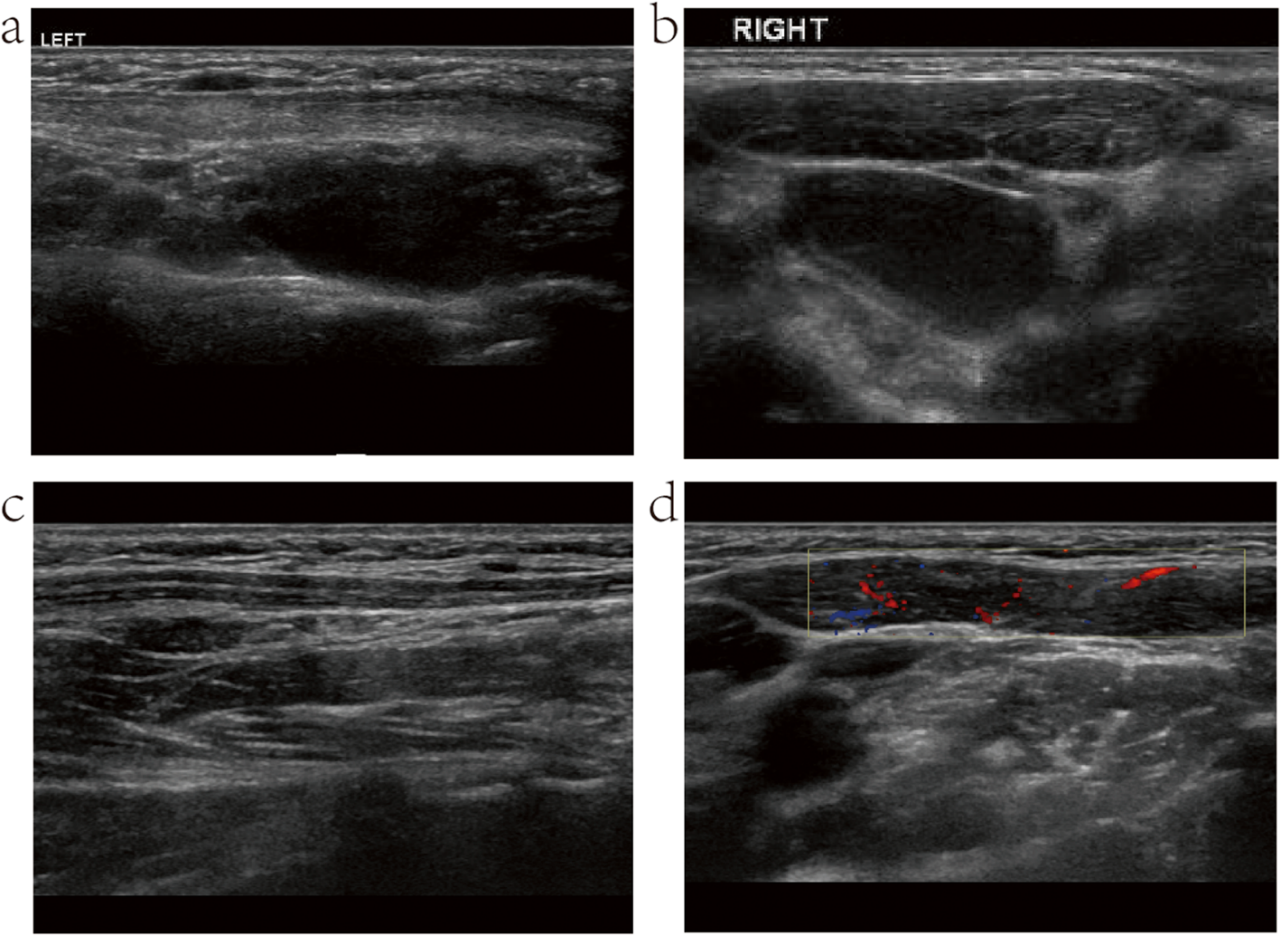
Ultrasound images of the two children in Case one and Case two at final follow-up (**a**) and (**b**) Ultrasound images of the involved and normal SCMs of the child in Case one at final follow-up, respectively, showing amyotrophy of the involved SCM (**c**) and (**d**) Ultrasound images of the involved and normal SCMs of the child in Case two at final follow-up, respectively, showing amyotrophy of the involved SCM

We followed up the children with MM for changes in the SCM, determined through physical examination (palpation and active and passive neck motion), ultrasonography (before MM, immediately after MM, and at final follow-up), and MRI. The results indicated an incomplete rupture of the SCM, possibly a rupture within the SCM mass or a local muscle tear. In addition, the final follow-up ultrasound images revealed a generally clear echotexture of the muscle fibers but with local thinning of the SCM, demonstrating the development of amyotrophy in patients with MM (Fig. 6).

So far, there are few reports on MM during physical treatment for CMT. The long-term effects of MM on the outcome of children with CMT and its influencing factors are unclear. There is no information on MM in the 2018 evidence-based clinical practice guideline for physical therapy management of CMT published by the American Physical Therapy Association [9]. This 5.5-year follow-up study demonstrated no particular deleterious effects of MM in children with CMT. Our findings can help physiotherapists understand MM in the process of physiotherapy, and provide a guide to standardized physiotherapy for infants with CMT.

#### Limitations

Events per variable are enough in the logistic regression analysis of this study. However, this study shows a limitation in its small sample from a single center, which may lead to selection bias. In subsequent studies, we will increase the sample size and add more objective parameters, such as quantification of craniofacial deformity changes, to reduce the subjective effects of doctors’ and guardians’ observation. Our team is preparing a prospective multi-center study with a large amount of detailed data. And we hope to use big medical data analysis to provide a new comprehensive individualized treatment strategy for CMT.

## Conclusion

MM may occur during physical therapy for infants with CMT, especially for newborns. It is a clinical phenomenon that occurs during physiotherapy, not an adverse event that may lead to a poor outcome. It is more likely to occur in younger children, those with a larger SCM mass, and with DDH. Physiotherapists should consider the definition and tetrad signs of MM, and carefully control the stretch strength to ensure effective treatment and also avoid severe complications. Children with MM generally have a good prognosis. However, we do not recommend MM as a goal of treatment. In clinical practice, physiotherapists should investigate relevant factors in advance to predict the possibility of MM, and take preventive measures to avoid possible complications. (Supplementary Material 4).

## Supplementary Information

**Additional file 1: Supplementary Figure 1.** Images of the child in Case two

**Additional file 2: Supplementary Figure 2.** Physical examination of the child in Case two after MM

**Additional file 3: Supplementary Figure 3. **Physical examination of the child in Case two at final follow-up

**Additional file 4:** **Supplementary Table 1. **Cheng-Tang rating scores between the MM and NMM groups

**Additional file 5:** **Supplementary material 1.** case one

**Additional file 6:** **Supplementary material 2. **case two

**Additional file 7:** **Supplementary material 4.**STROBE checklist

**Additional file 8.** STROBE Statement—Checklist of items that should be included in reports of cohort s

## Data Availability

The datasets generated and/or analyzed during the current study are not publicly available due to limitations of ethical approval involving the patient data and anonymity but are available from the corresponding author on reasonable request.

## Abbreviations

CMT: Congenital muscular torticollis
DDH: Developmental dysplasia of the hip
SCM: Sternocleidomastoid muscle
MM: Manual myotomy
ECM: Extracellular matrix
