# Supplementary figures and images for: Factors influencing and long-term effects of manual myotomy phenomenon during physiotherapy for congenital muscular torticollis
Source: BMC Musculoskelet Disord. 2022 Oct 1;23:892. doi: 10.1186/s12891-022-05788-7 (PMC9526270; doi:10.1186/s12891-022-05788-7)

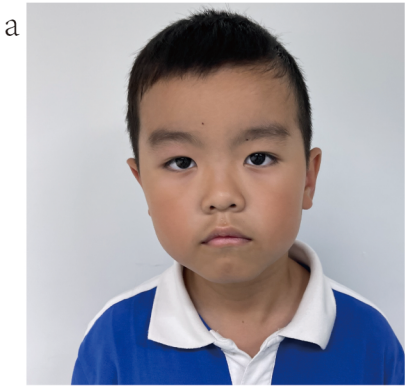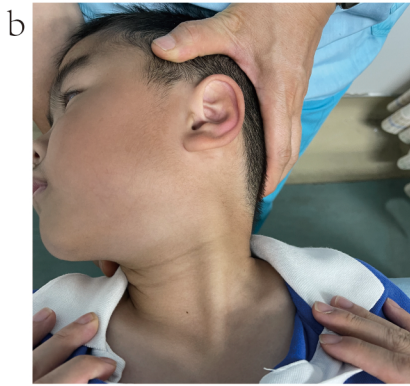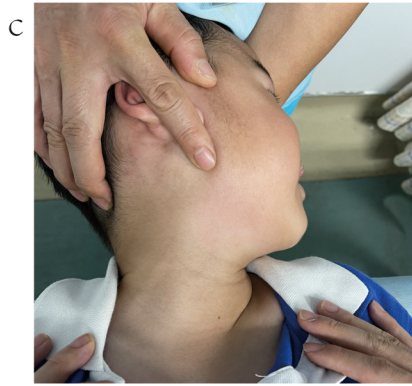

Supplement: Supplementary file 1 — Additional file 1: Supplementary Figure 1. Images of the child in Case two [file 12891_2022_5788_MOESM1_ESM.pdf]

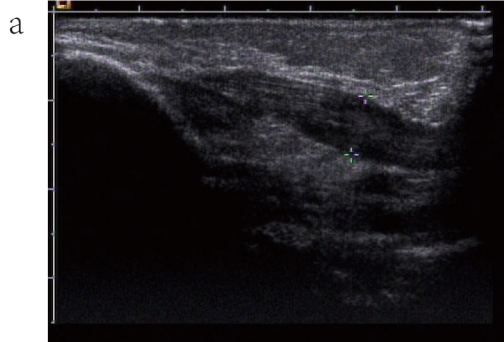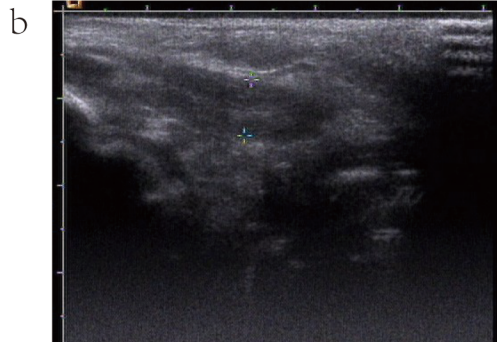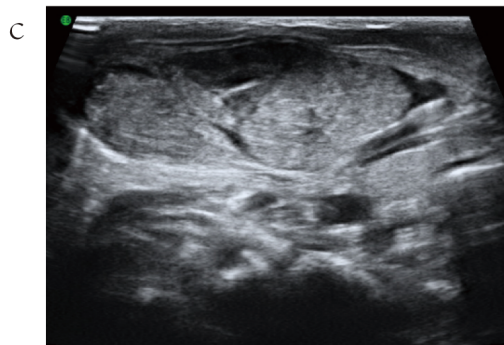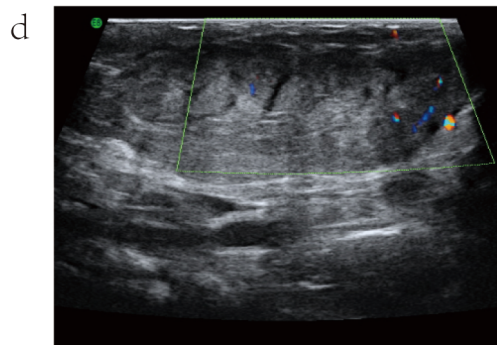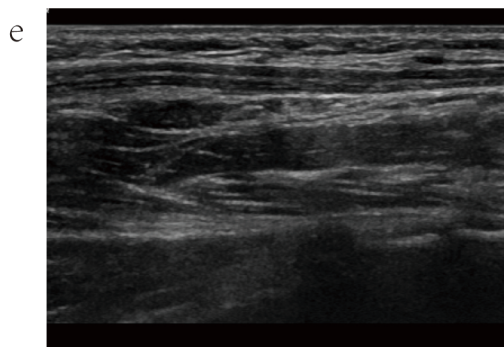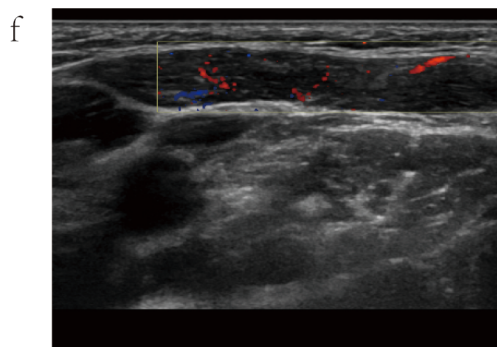

Supplement: Supplementary file 4 — Additional file 4: Supplementary Table 1. Cheng-Tang rating scores between the MM and NMM groups [file 12891_2022_5788_MOESM4_ESM.pdf]

a

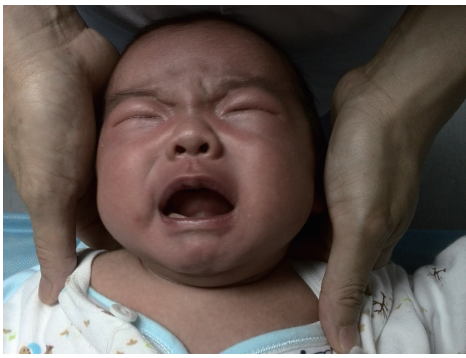

b

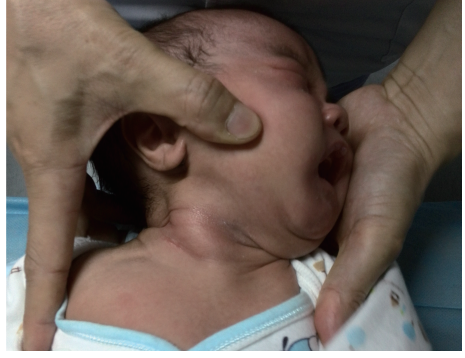

c

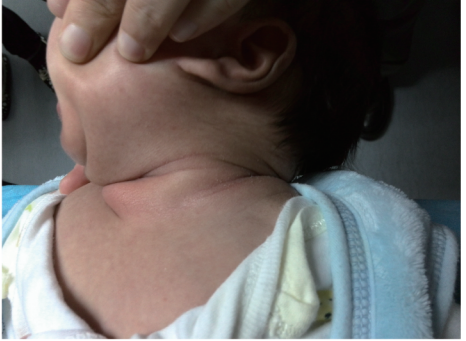

d

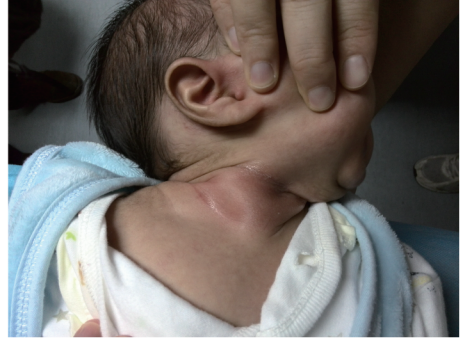

Supplement: Supplementary file 5 — Additional file 5: Supplementary material 1. case one [file 12891_2022_5788_MOESM5_ESM.pdf]
