# Supplementary material for: Factors influencing and long-term effects of manual myotomy phenomenon during physiotherapy for congenital muscular torticollis
Source: BMC Musculoskelet Disord. 2022 Oct 1;23:892. doi: 10.1186/s12891-022-05788-7 (PMC9526270; doi:10.1186/s12891-022-05788-7)
Supplement: Supplementary file 2 — Additional file 2: Supplementary Figure 2. Physical examination of the child in Case two after MM [file 12891_2022_5788_MOESM2_ESM.docx]

**Supplementary Figure 1:** Images of the child in Case two

(a) and (b) Ultrasound images of the involved and normal SCMs of the child at initial visit

(c) and (d) Ultrasound images of the involved and normal SCMs of the child immediately after MM

(e) and (f) Ultrasound images of the involved and normal SCMs of the child at final follow-up

**Supplementary Figure 2:** Physical examination of the child in Case two after MM

(a) Front view of the infant

(b) View of the mass

(c) View of neck rotation to the involved side

(d) View of neck rotation to the normal side

Note: the red triangle represents the location of the SCM mass

**Supplementary Figure 3:** Physical examination of the child in Case two at final follow-up

(a) Front view of the infant

(b) View of neck rotation to the involved side

(c) View of neck rotation to the normal side

**Note:** the red triangle represents the location of the SCM mass

**Supplementary Table 1:** Cheng-Tang rating scores between the MM and NMM groups

**Supplementary material 1:** case one

**Supplementary material 2:** case two

**Supplementary material 4:** STROBE checklist
