# Supplementary material for: Factors influencing and long-term effects of manual myotomy phenomenon during physiotherapy for congenital muscular torticollis
Source: BMC Musculoskelet Disord. 2022 Oct 1;23:892. doi: 10.1186/s12891-022-05788-7 (PMC9526270; doi:10.1186/s12891-022-05788-7)
Supplement: Supplementary file 6 — Additional file 6: Supplementary material 2. case two [file 12891_2022_5788_MOESM6_ESM.docx]

We present a case of congenital muscular torticollis (CMT) in a participant who developed manual myotomy (MM) during treatment with manual stretching. We followed up this child until publication of this paper, and relevant clinical and imaging data are described below. The child was delivered in the left sacrum anterior position through caesarean section with breech traction by a gravida 4 para 1 mother. The child weighed 3.58 kg and was 51 cm long at birth, with clear amniotic fluid and a single loop of umbilical cord around the neck. The Apgar scores at one minute and five minutes after birth were 10 points. Physical examination was normal.

On day 14 after birth, a hard, fixed, non-tender mass of about 4×3×2 cm was palpated on the left side of the neck. The head was slightly tilted to the left side and the face was generally symmetrical. There was significant limitation in the range of lateral flexion and rotation of the neck. Ultrasonography showed thickening of the left sternocleidomastoid muscle (SCM) and a nodular appearance of the middle and lower portions of the muscle (of about 2.7×2.3×1.1 cm). There was also enhanced and inhomogeneous echogenicity and a disorganized and unclear muscle fiber echotexture. Finally, there was no evident local thickening of the right SCM (the upper portion 0.31 cm, middle portion 0.32 cm, and lower portion 0.33 cm in thickness), and this had normal echogenicity and a clear muscle fiber echotexture. The patient was diagnosed with left CMT.

On day 17 after birth, the patient was referred to our hospital to receive manual stretching. During treatment, MM occurred and the infant presented with swelling and tenderness in the mastoid portion of the left SCM and reduced limitation in the range of neck rotation and side flexion. The infant had normal consciousness, responsiveness, and breathing without vomiting. Immediate ultrasound scans showed nodular thickening (about 2.8×2.5×1.2 cm) of the middle portion of the left SCM, with enhanced and inhomogeneous echogenicity and a disorganized and unclear muscle fiber echotexture; and inhomogeneous echogenicity of the lower sternal head of the left SCM, about 2.3 cm in length and 0.4 cm in thickness, containing discrete dark echoes with a maximum range of about 0.5×0.3 cm. There was normal echogenicity and a clear muscle fiber echotexture of the right SCM (the thicknesses of the upper, middle, and lower portions were 0.31 cm, 0.32 cm, and 0.33 cm, respectively), without evident local thickening or space-occupying echoes. MRI showed swelling and a tear of the left SCM, with ring edema surrounding it. Three days later, physical examination revealed reduced swelling and tenderness in the mastoid portion of the left SCM, and the previous limitations in the range of neck rotation and side flexion were significantly resolved compared with the status before MM (**Figures 2** and **3**).

After the swelling of the mastoid portion of the left SCM disappeared, the infant resumed manual stretching at our hospital, in combination with ultrasonic physiotherapy. More than one month after MM, the face was generally symmetrical; there was no evident head tilting; and the limitation in the range of neck rotation and side flexion was ameliorated. Ultrasonography showed a slight thickening of the middle portion of the left SCM, about 2.8×0.9×0.6 cm in size, with enhanced and slightly inhomogeneous echogenicity and a disorganized and unclear muscle fiber echotexture. There was slight inhomogeneous echogenicity of the lower sternal head of the SCM, about 2.0 cm in length and 0.5 cm in thickness, containing small discrete patches that were extremely hypoechoic; and normal echogenicity and a clear muscle fiber echotexture of the right SCM (the thicknesses of the upper, middle, and lower portions were 0.31 cm, 0.32 cm, and 0.33 cm, respectively), without evident local thickening or space-occupying echoes.

Two and a half years after MM, the child revisited our hospital and had no evident abnormalities on physical examination, with a generally symmetrical face and without evident head titling or limited neck rotation or side flexion. Ultrasonography showed thinning of the middle and lower portions of the left SCM, especially for the sternal head, with slight echo enhancement (about 0.3 cm in thickness) and a generally clear muscle fiber echotexture. There was normal echogenicity and a clear muscle fiber echotexture of the right SCM (the thicknesses of the upper, middle, and lower portions were 0.48 cm, 0.49 cm, and 0.49 cm, respectively), without evident local thickening or space-occupying echoes (**Figure 4**).

During the 2.5 year follow-up, the child had no special complications except initial swelling and tenderness in the mastoid portion of the left SCM, with a relatively short time to disappearance of the SCM mass and good improvement of the range of motion in neck rotation and side flexion. The child had a good outcome.
