# Supplementary material for: Factors influencing and long-term effects of manual myotomy phenomenon during physiotherapy for congenital muscular torticollis
Source: BMC Musculoskelet Disord. 2022 Oct 1;23:892. doi: 10.1186/s12891-022-05788-7 (PMC9526270; doi:10.1186/s12891-022-05788-7)
Supplement: Supplementary file 7 — Additional file 7: Supplementary material 4.STROBE checklist [file 12891_2022_5788_MOESM7_ESM.docx]

In a previous screening study of 2564 full-term (> 37 weeks) Chinese newborns for congenital muscular torticollis (CMT) ^[4]^, our team shared a case of CMT who received comprehensive professional physical treatment at our hospital and developed manual myotomy (MM). We followed up the child for seven years, and found that after treatment, the child recovered free rotation and side flexion of the neck, and had no special abnormalities except an acceptable mild head tilt. We provide a detailed description of the diagnosis and treatment of the case to demonstrate our opinions.

The child was born through caesarean section by a gravida 2 para 1 mother, weighing 3.78 kg and was 51 cm long at birth, with clear amniotic fluid and 1- and 5-minute Apgar scores of 10 points. Physical examination one day after birth revealed facial asymmetry (the right side of the face was smaller than the left) and tightness of the left sternocleidomastoid muscle (SCM). There was no palpable tumor in the neck. The range of motion of the neck was normal.

On the first day after birth, ultrasonography showed changes in the echotexture of the bilateral SCMs (the right SCM was 0.48 cm thick and the left SCM 0.68 cm thick), without evidence of tumor echoes. On day 7, there was no evident mass in the SCM on physical examination, and ultrasound found that the right SCM was 0.62 cm thick and the left SCM 0.65 cm thick. On day 15, physical examination revealed obvious facial asymmetry (the right side of the face was smaller than the left), the head was tilted to the right side, and a tumor 3.0×2.0×1.0 cm was visible in the right SCM. Ultrasound showed hyperechoic thickening of the right SCM (the middle portion was 0.64 cm in thickness); and normal echogenicity and a clear muscle fiber echotexture of the left SCM (the upper, middle, and lower portions were 0.41 cm, 0.43 cm, and 0.42 cm in thickness, respectively), without evident local thickening or space-occupying echoes. On day 22, the child had obvious facial asymmetry and limited rotation and side flexion of the neck on physical examination. Ultrasound showed hyperechoic thickening of the entire right SCM (the upper, middle, and lower portions were 0.81 cm, 0.93 cm, and 0.85 cm in maximum thickness, respectively). There was normal echogenicity and a clear muscle fiber echotexture of the left SCM (the upper, middle, and lower portions were 0.42 cm, 0.43 cm, and 0.42 cm in thickness, respectively), without evident local thickening or space-occupying echoes.

About one month after birth, the child developed MM during treatment with manual stretching at our hospital. Instant ultrasonography found hyperechoic thickening of the right SCM containing small discrete patches that were extremely hypoechoic (the upper portion was 0.66 cm in maximum thickness with a disorganized and slightly inhomogeneous muscle fiber echotexture, the middle portion 1.24 cm in maximum thickness, and the lower portion 1.00 cm in maximum thickness). There were small fluid sonolucent areas surrounding the right SCM, with a maximum depth of 0.5 cm; and normal echogenicity and a clear muscle fiber echotexture of the left SCM (the upper, middle, and lower portions were 0.43 cm, 0.44 cm, and 0.43 cm in thickness, respectively), without evident local thickening or space-occupying echoes (**Supplementary Figure 2**).

At 3 years and 8 months of age, the child had no evident abnormalities on physical examination, with free rotation and side flexion of the neck. Ultrasound showed diffuse thinning of the right SCM (especially for the sternal head; the middle-lower portion being about 0.15 cm in minimum thickness), with slightly inhomogeneous internal echoes and a generally clear muscle fiber echotexture. There was homogeneous echogenicity of the left SCM, with the upper, middle, and lower portions being 0.46 cm, 0.47 cm, and 0.47 cm in thickness, respectively.

At 4 years and 11 months of age, there were no evident abnormalities on physical examination, with free rotation and side flexion of the neck. Ultrasound showed diffuse thinning of the right SCM (especially for the sternal head; with the upper, middle, and lower portions being 0.43 cm, 0.28 cm, and 0.17 cm in thickness, respectively), with slightly inhomogeneous internal echoes and a generally clear muscle fiber echotexture. There was homogeneous echogenicity of the left SCM, with the upper, middle, and lower portions being 0.45 cm, 0.46 cm, and 0.46 cm in thickness, respectively.

At 7 years of age, the child had no special abnormalities except a mild head tilt, without limitation in the range of neck rotation and side flexion (**Supplementary Figure 3**). Ultrasound showed thinning of the right SCM (the upper, middle, and lower portions being 0.27 cm, 0.24 cm, and 0.20 cm in thickness, respectively), with generally homogeneous internal echoes and a generally clear muscle fiber echotexture. There was homogeneous echogenicity of the left SCM, with the upper, middle, and lower portions being 0.43 cm, 0.44 cm, and 0.42 cm in thickness, respectively. Through the detailed follow-up records with physical examination and ultrasonography (**Supplementary Figure 1**), we believe that the child has a satisfactory outcome on the whole. We will continue the follow-up for further long-term information.
