# Supplementary material for: Factors influencing and long-term effects of manual myotomy phenomenon during physiotherapy for congenital muscular torticollis
Source: BMC Musculoskelet Disord. 2022 Oct 1;23:892. doi: 10.1186/s12891-022-05788-7 (PMC9526270; doi:10.1186/s12891-022-05788-7)
Supplement: Supplementary file 8 — Additional file 8. STROBE Statement—Checklist of items that should be included in reports of cohort s [file 12891_2022_5788_MOESM8_ESM.docx]

| Supplementary Table 1 Cheng-Tang rating scores between the MM and NMM groups | | | | | |
| --- | --- | --- | --- | --- | --- |
| Item | Group | Excellent N | Good N | Fair N | P |
| Rotation angle score | MM group | 41 | 0 | 0 | _ |
|  | NMM group | 48 | 0 | 0 |  |
| Side flexion score | MM group | 41 | 0 | 0 | _ |
|  | NMM group | 48 | 0 | 0 |  |
| Contracture muscle bundle | MM group | 41 | 0 | 0 | 0.059 |
|  | NMM group | 45 | 0 | 3 |  |
| Stiffness of SCM mass | MM group | 40 | 0 | 1 | 0.461 |
|  | NMM group | 48 | 0 | 0 |  |
| Active head tilt | MM group | 41 | 0 | 0 | 0.246 |
|  | NMM group | 44 | 1 | 3 |  |
| Head tilt while sleeping | MM group | 40 | 0 | 1 | 0.212 |
|  | NMM group | 43 | 0 | 5 |  |
| Daily activities | MM group | 40 | 0 | 1 | 0.407 |
|  | NMM group | 46 | 0 | 2 |  |
| Improvement in face size | MM group | 39 | 0 | 2 | 0.145 |
|  | NMM group | 39 | 2 | 7 |  |
| Head shape | MM group | 36 | 0 | 5 | 0.396 |
|  | NMM group | 38 | 0 | 10 |  |
| Head improvement | MM group | 38 | 0 | 3 | 0.301 |
|  | NMM group | 44 | 0 | 4 |  |
| Subjective head tilt | MM group | 41 | 0 | 0 | < 0.05 |
|  | NMM group | 25 | 3 | 20 |  |
